# INTERNATIONAL LONG-TERM CARE HOME QUALITY ASSURANCE: HOW DO EUROPEAN MEASURES ADDRESS DEMENTIA?

**DOI:** 10.1093/geroni/igac059.2803

**Published:** 2022-12-20

**Authors:** Michael Lepore, Franziska Zúñiga, Jos Schols, Klaus Wingenfeld, Theo van Achterberg, Briana Murray

**Affiliations:** University of Maryland School of Nursing, Baltimore, Maryland, United States; University of Basel, Basel, Basel-Stadt, Switzerland; Maastricht University, Maastricht, Limburg, Netherlands; University of Bielefeld, Bielefeld, Nordrhein-Westfalen, Germany; KU Leuven, Leuven, Vlaams-Brabant, Belgium; University of Maryland School of Nursing, Baltimore, Maryland, United States

## Abstract

Alzheimer’s Disease and related dementias (ADRD) are prevalent conditions in long-term care homes (LTCHs) with about 50% of LTCH residents living with ADRD in many countries. Despite the prevalence of ADRD in LTCHs, a recent examination of LTCH quality assurance programs in four countries revealed a small percentage of LTCH quality measures across those countries addressed ADRD, most commonly as a risk adjuster, and once each as an inclusion or exclusion criterion. To better understand how quality assurance programs address ADRD internationally, we examined LTCH quality measures in four European countries—The Netherlands, Switzerland, Germany, and Belgium—following procedures previously used to examine international LTCH quality assurance measures. 73 measures were examined across 4 quality assurance programs. 26% of the measures addressed ADRD. The programs addressed ADRD in starkly different ways: in The Netherlands and Belgium no measures addressed ADRD; in Germany the majority (13/15) of measures addressed ADRD as an exclusion or inclusion criterion; and in Switzerland all the measures addressed ADRD through risk adjustment. Although limited to examining measures from LTCH quality assurance programs in four European countries, this study adds evidence that ADRD tends not to be addressed by LTCH quality measures, but when ADRD is addressed, it tends to be through risk adjustment. LTCH regulators, policymakers, and providers can use this information to assess options for addressing ADRD in quality assurance programs. Future research is needed to assess how standard indicators of quality differ across quality assurance regimes and how to address dementia beyond risk adjustment.

